# Effect of repetition of vertical and horizontal routes on navigation performance in Australian bull ants

**DOI:** 10.3758/s13420-023-00614-z

**Published:** 2023-12-05

**Authors:** Vito A. G. Lionetti, Ken Cheng, Trevor Murray

**Affiliations:** School of Natural Sciences, Macquarie University, Sydney, NSW 2109 Australia

**Keywords:** *Myrmecia midas*, Navigation, Path integration, View-based navigation, Ants

## Abstract

Solitarily foraging ant species differ in their reliance on their two primary navigational systems— path integration and visual learning. Despite many species of Australian bull ants spending most of their foraging time on their foraging tree, little is known about the use of these systems while climbing. “Rewinding” displacements are commonly used to understand navigational system usage, and work by introducing a mismatch between these navigational systems, by displacing foragers after they have run-down their path integration vector. We used rewinding to test the role of path integration on the arboreal and terrestrial navigation of *M. midas*. We rewound foragers along either the vertical portion, the ground surface portion, or across both portions of their homing trip. Since rewinding involves repeatedly capturing and releasing foragers, we included a nondisplacement, capture-and-release control, in which the path integration vector is unchanged. We found that rewound foragers do not seem to accumulate path integration vector, although a limited effect of vertical rewinding was found, suggesting a potential higher sensitivity while descending the foraging tree. However, the decrease in navigational efficiency due to capture was larger than the vertical rewinding effect, which along with the negative impact of the vertical surface, and an interaction between capture and rewinding, may suggest aversion rather than path integration caused the vertical rewinding response. Together these results add to the evidence that *M. midas* makes minimal use of path integration while foraging, and the growing evidence that they are capable of quickly learning from aversive stimulus.

**Supplementary Information:**

The online version contains supplementary material available at 10.3758/s13420-023-00614-z.

## Introduction

Insect nervous systems utilize a combination of multiple navigational systems, some of which require learning. To return successfully to the nest after a foraging journey, solitarily foraging ants can combine odometric and compass information into a vector, with a neural mechanism called *path integration* (Webb, 2019; Wehner & Wehner, 1986). In addition, many ant species use terrestrial landmarks, which once learned provide local navigational guidance (Cheng et al., 2009; Freas et al., 2017a, b; Warrant & Dacke, 2010). Many solitarily foraging ants rely simultaneously on path integration and vision-based navigation systems to find their way home (Hoinville & Wehner, 2018; Wehner et al., 2016). Although redundant, the navigational guidance of terrestrial cues and path integration provides robustness (Heinze et al., 2018). The errors accumulated during path integration and changes in panoramas from what was learned may each cause the foragers to fail in reaching the nest (Collett, 2012; Narendra, 2007). And so, by integrating the outputs of these two navigation systems, ants can overcome errors that would otherwise lead them astray (Collett, 2012; Legge et al., 2014; Narendra, 2007; Wehner et al., 2016).

Solitarily foraging ants differ in the extent to which they rely on path integration and terrestrial cues (Cheng et al., 2014). Desert ants living in landmark-rich environments, such as *Melophorus bagoti*, rely more on terrestrial cues than do desert ants living in landmark-meagre environments (Bühlmann et al., 2011; Cheng et al., 2014). Australian bull ants, such as *Myrmecia midas,* which live in feature-rich forest and periphery environments, rely heavily on visual cues, and exhibit minimal use of path integration (Freas et al., 2017a, b).

While species differ in their baseline utilization of path integration and terrestrial cues, individual foragers can modify the weight given to navigational cues and path integration according to their individual context and experience (Wystrach et al., 2020). When foragers find themselves in a visually unfamiliar place, either through travel beyond their normal range, or a sudden change in the scene (such as a tree fall), foragers appear to evaluate the reliability of both navigational systems to determine their behavioural response (Narendra et al., 2013; Wehner et al., 2016). In comparing navigational information, foragers give more weight to path integration vectors as they increase in length, such as after travelling a longer path (Hoinville & Wehner, 2018; Wystrach et al., 2015), and more weight to familiar and more recently experienced views (Freas & Cheng, 2017). In addition, desert ants reduce the weights given to landmark cues along the route when they have failed to reach their intended destination on previous trips (Andel & Wehner, 2004; Collett, 2014; Wystrach et al., 2019). Overall, many foraging ants have the ability to adjust the weight given to their navigational systems based on their individual context and experience.

However, these weights are usually hard to measure and understand because, in natural conditions, all sources of navigational information are nearly in accord. Instead, we must introduce conflict between these systems, such as through rewinding (Wystrach et al., 2019). During rewinding, homing foragers re-experience a portion of their foraging route in such a way as to add a vector in the opposite nest-to-foraging tree direction without changing the direction indicated by visual scene cues (Wystrach et al., 2019). As such, the vector can be zeroed by catching them as they first approach the nest; further rewinding then extends the vector in the direction opposite the homing direction since the vector continues to accumulate in the opposite direction to the one travelled. Such a rewound vector indicates a fictive goal located at the feeder location, and then, with further rewinding, beyond that starting point. Such rewinding procedures cause desert ant foragers to exhibit higher meandering and more scanning behaviours when homing after being rewound (Andel & Wehner, 2004; Wystrach et al., 2019) and increase the proportion of these ants that perform U-turns in the direction of the nest-to-feeder vector (Collett, 2014; Wystrach et al., 2019).

One often unaddressed issue with this experimental design is that the rewinding procedure makes the foragers experience unsuccessful homing trips, and involves capturing, each of which could be perceived as aversive events. These unsuccessful homing trips could decrease foragers’ confidence and willingness to use the terrestrial cues, even those that had previously led successfully to the nest (Wystrach et al., 2019). Some species, such as *M. midas* foragers even respond to the physical manipulation of the capture as if it is aversive, by circumventing the capture location and increasing both meandering and scanning behaviour (Lionetti et al., 2023). However, other species, such as *M. bagoti* foragers, do not show any aversive responses despite being captured multiple times (Wittlinger et al., 2006; Wystrach et al., 2019).

It is unclear how foragers walking on a tree might integrate path integration and terrestrial cues after being rewound. Most navigational research on ants has focused on horizontal navigation, and little is known about their vertical navigation (but see Freas et al., 2018). *Myrmecia midas* foragers use panorama cues to orient successfully while descending from the foraging tree: They move during their descent to the side of the tree where their nest is located (Freas et al., 2018). Additionally, *M. midas* foragers use terrestrial cues to avoid barriers by taking alternative paths during their vertical ascent on foraging trips (Islam et al., 2023). Rewound over the horizontal foraging corridor, *M. midas* foragers relied on terrestrial cues even after nine rewinding procedures, although they exhibited an increase in meandering and scanning behaviours, and occasional brief U-turns shorter than 2% of the vector length (Deeti et al., 2023). Nothing is known, however, about how they integrate path integration and terrestrial cues during vertical navigation. Previous research showed that *Cataglyphis fortis* foragers do not compute their path integration in three dimensions. *Cataglyphis fortis*, however, is known to navigate solely in a two-dimensional environment (Ronacher, 2020). The two-dimensional nature of path integration is supported by insects’ central complex, in which path integration compass directions are supported by eight TB1 cells, which tune different neuronal inputs into one specific azimuth direction output (Heinze et al., 2018; Stone et al., 2017). In contrast, *M. midas* foragers are known to navigate on foraging trees where they spend the majority of their foraging time (Freas et al., 2018).

Since it is not known whether foragers accumulate a path integration vector while traveling vertically on a tree, we compared the effect of vertical and horizontal rewinding on their navigational performance. Since rewinding works by allowing foragers to run down and then accumulate a vector through their own motion, we can use it to introduce conflict between the path integration vector and the visual scene. As such, we rewound foragers during the vertical (Rewind Tree), and horizontal (Rewind Terrain) of portions of their homing trip. We also rewound ants across their entire foraging corridor, vertical and horizontal (Rewind Trip). We checked for changes in three aspects of their navigational behaviour: their meandering, U-turns, and scanning rate. Since rewinding involves capturing and displacing foragers, which some species find aversive (Lionetti et al., 2023), we included a control for capturing and releasing them at the point of capture. We also investigated differences in their navigational behaviour due to moving vertically on a tree versus horizontally across the ground. Finally, we investigated whether path integration and terrestrial navigation have a similar contribution to the foragers’ initial meandering and angular velocity as in their whole homing trip. If foragers only accumulate a path integration vector while traveling horizontally, we predict that Rewind Terrain and Rewind Trip foragers would exhibit worse navigational behaviour than Rewind Tree foragers. In contrast, if foragers also accumulate a vector on the tree, we expect Rewind Tree and Terrain to be the same but may expect Rewind Trip foragers to have the most disrupted navigational behaviour due to accumulating along both the vertical and horizontal segments. Together we hope these experiments help us to understand *M. midas*’s use of path integration, its role in vertical walking, and the effect of capture and release on their navigational behaviour.

## Method

### Field site and study species

We conducted two experiments on two different *M. midas* nests located on the Macquarie University campus, Sydney, Australia. *Myrmecia midas* nests are usually located close to the base of an *Eucalyptus* tree. As well as the adjacent ‘nest’ tree, *M. midas* ants often forage on other nearby (3–8 meters) *Eucalyptus* trees, commonly called foraging trees. *Myrmecia midas*’s foraging activity starts during evening twilight, with foragers returning to the nest during morning twilight (Freas et al., 2017a, b). We conducted the first experiment between November 2019 and January 2020, and the second experiment between February and April 2022. The nests were located in an area with *Eucalyptus* trees, grassy surroundings, and buildings. No ethical approval was necessary in Australia to test ants, and all the manipulations and tests were noninvasive, producing no lasting adverse effects on the ants or nests.

### Test site and experimental setup

Each of the two test sites consisted of two sections, the Horizontal section, which was the ground area where most of the foragers’ trips occurred, and the Vertical section, which was a section of the foraging tree including the foragers’ foraging paths. The Horizontal section measured 4 × 3 meters and contained the nest and the base of the foraging tree. The Vertical section was 2 meters high and 1.2 meters wide around the tree’s circumference, which we subdivided into 10-centimetres squares. We removed superficial dead vegetation in the terrain section to improve observer visibility. We subdivided the Horizontal section into 50 centimetres squares by using string and tent pegs. We allowed all foragers to forage on the foraging tree for 2 days to habituate them to the terrain changes due to vegetation removal. We then caught each forager during their first appearance on the foraging tree using a plastic vial. We induced a chill coma by placing the ant in a box of ice for 5 minutes, allowing us to mark the forager with an individual combination of different paint colours (Tamiya ^TM^). We provided the ant with a small amount of sugar water in the vial, which we placed in a darkened box until the next morning between 7:00 and 9:00 a.m., when we released them to return home from the foraging tree. We used a red-light headlamp to aid with visibility during these post-sunset activities.

### Experimental procedure

We captured each marked forager upon her next appearance using a plastic vial during her evening foraging outbound trip, between 20:15 and 21:15, on the foraging tree at 1.5 meters in height. We fed the forager with a small amount of honey water and confined them until the following morning. Between 06:30 and 09:30, we released the forager at the foraging tree’s release point, which was 1.5 meters high and facing the nest direction. The forager was then allowed to return to the nest. During the first inbound trip, called the 0^th^ trial, the treatment forager was captured in the proximity of the nest and released at different locations depending on test conditions. The treatment forager underwent a rewinding procedure, which involved being captured and released on the next four inbound trips (1^st^ to 4^th^ trips). The control foragers did not undergo a rewinding procedure (see Test Conditions). On the 4^th^ trip, we allowed the forager to return to the nest, ending its participation in the test. We marked the foragers with a specific colour at the end of the experiment to help us recognize the already-tested foragers.

### Test conditions

**Fig. 1:**
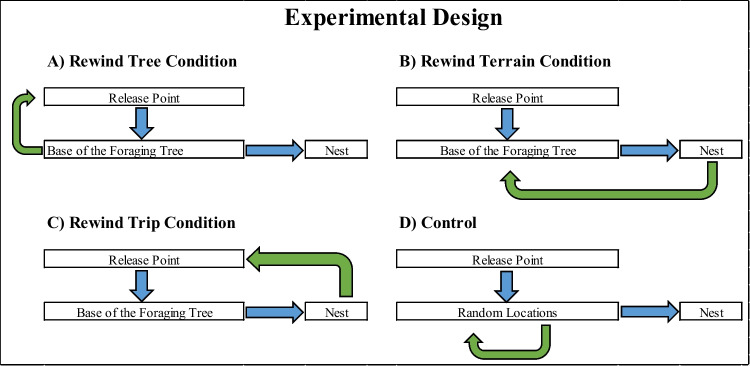
Flow chart of the experimental design. **A)** In the Rewind Tree condition, a forager was displaced to the release point once it reached the base of the foraging tree. The procedure was repeated 3 times. **B)** In the Rewind Terrain condition, a forager was displaced to the base of the foraging tree once it reached the nest location. The procedure was repeated 3 times. **C)** In the Rewind Trip condition, a forager was displaced to the release point once it reached the nest location. The procedure was repeated 3 times. **D)** In the Control condition, a forager was captured at random locations and released at the same locations. This procedure was repeated for 3 more trips. (Colour figure online)

To compare the effects of horizontal and vertical rewinding we tested the foragers in four conditions: Rewind Terrain, Rewind Tree, Rewind Trip, and Control (for each condition, *n* = 15) (Fig. 1). Foragers were randomly assigned to one of the four test conditions. In the Rewind Tree condition, the homing foragers were captured just before the end of the tree section and displaced back at the release point, on the foraging tree at 1.5 meters height, allowing them to reexperience the path on the foraging tree (Fig. 1A). In the Rewind Terrain condition, the foragers were captured near the nest and displaced to the base of the foraging tree, reexperiencing the inbound trip occurring on the ground, 2.5 meters long (Fig. 1B). In the Rewind Trip condition, the foragers were captured near the nest and displaced at the release point on the tree, allowing them to reexperience the full inbound path (Fig. 1C). In the Control condition, the foragers experienced three interruptions: they were captured from randomly chosen locations, held for 10 seconds, and then released at the same spot during their 1^st^ homing trip (Fig. 1D).

### Data analysis

#### Experiment 1

We investigated the contribution of path integration and terrestrial navigation systems on homing foragers while walking on the foraging tree or the foraging corridor. We recorded the foragers’ path, U-turns, and scanning behaviours using gridded paper (*n* = 15 for each condition). We used Web Plot Digitizer (https://automeris.io/WebPlotDigitizer/) to digitize the scanned paths into *x–y* coordinates. We use the software R to process and analyze the data collected (R Core Team, 2021). We calculated the meandering using the formula: Sinuosity = 2[p (((1 + c) / (1 − c)) + b^2)] ^−0.5^, where c is the mean cosine of turning angles, *p* and b are the expectation and the coefficient of variation of the step length (Benhamou, 2004). We used every recorded data fragment as a step length for the expectation and the coefficient of variation. The sinuosity obtained by this function ranges between 0, a straight path, and 1, a highly curved path. Since ants vary in the length of their trip, we calculated the U-turn and scanning rate as a proportion of their distance travelled. The U-turn rate is the number of times foragers turned around and began travelling in nest-to-foraging-site direction for more than 10 centimetres, divided by the length of the path travelled in centimetres. We defined a scan as a forager stopping forward movement and performing saccadic body rotations at one spot, known as a scanning bout. We considered each individual scanning bout as one scan. We calculated the scanning rate by counting the number of scans, divided by the total path travelled in centimetres.

#### Experiment 2

We investigated whether path integration and terrestrial navigation have a similar contribution to the foragers’ initial navigation as in their whole homing trip. We recorded the ants’ initial meandering and initial absolute angular velocity using a high-resolution camera positioned on a tripod (3,840 × 2,160 pixels, Sony FDR-AX100E) (*n* = 37). We recorded an area of 30 centimetres^2^ and 60 centimetres^2^ in the Vertical and Horizontal sections, respectively. Due to logistical difficulties in the Control condition, we could not record it with a camera. To extract and analyze the forager’s body position from video recordings, we used DeepLabCut (Mathis et al., 2018; Nath et al., 2019) and MATLAB (MathWorks, Natick, MA, USA). We extracted six body positions: mandible tips, left compound eye, right compound eye, back head, front thorax, and abdominal petiole (Fig. S1). We calculated the initial absolute angular velocity by the rate of angular position changes over time (sampling rate 0.04 s) of the line connecting the foragers’ back head to their mandible tips.

### Statistical analyses

For each experiment, we used three groups of models (1, 2, and 3) to analyze our hand-recorded dataset from Experiment 1, and two groups of models (4 and 5) for our video-recorded dataset from Experiment 2. Model Groups 1 and 4 analyze the Vertical sections of the dataset, Groups 2 and 5 analyze the Horizontal sections, and Group 3 compares between Vertical and Horizontal sections. In the first and second models, we examined the effect of our treatment Conditions and the number of Captures on meandering, U-turn rate, and scanning rate in the Vertical and Horizontal sections, respectively (Experiment 1). For the treatment conditions, we defined the number of captures as the number of rewinding procedures. Whereas for the Control condition, we used the locations of capture interruptions to segment the homing trip into distinct portions, allowing us to observe foragers’ behaviours under varying numbers of experienced captures. In the third group of models, we tested for differences in behaviour in the Vertical and Horizontal sections in terms of meandering, U-turn, and scanning rate (Experiment 1). In the fourth and fifth model groups, we examined the effect of our treatment Conditions and the number of trips on initial meandering and initial scanning rate in the Vertical and Horizontal sections, respectively (Experiment 2). Before constructing the models we removed outliers, values exceeding two standard deviations, from the dataset and used Shapiro–Wilk normality test to test normality of meandering (W = 0.99, *p* ≤ .01), U-turns (W = 0.58, *p* ≤ .01), scanning rate (W = 0.59, *p* ≤ .01), initial meandering (W = 0.74, *p* ≤ .01), and initial scanning rate (W = 0.96, *p* ≤ .01) (Shapiro & Wilk, 1965). In every model, we used a generalized linear mixed-model analysis of variance (family Gaussian) with ant ID as a random effect (ANOVA) with *p* = .01 as an alpha level. We performed post hoc comparisons using Tukey tests with *p* = .01 as the alpha level.

## Results

### Experiment 1: Effect of rewinding

**Fig. 2:**
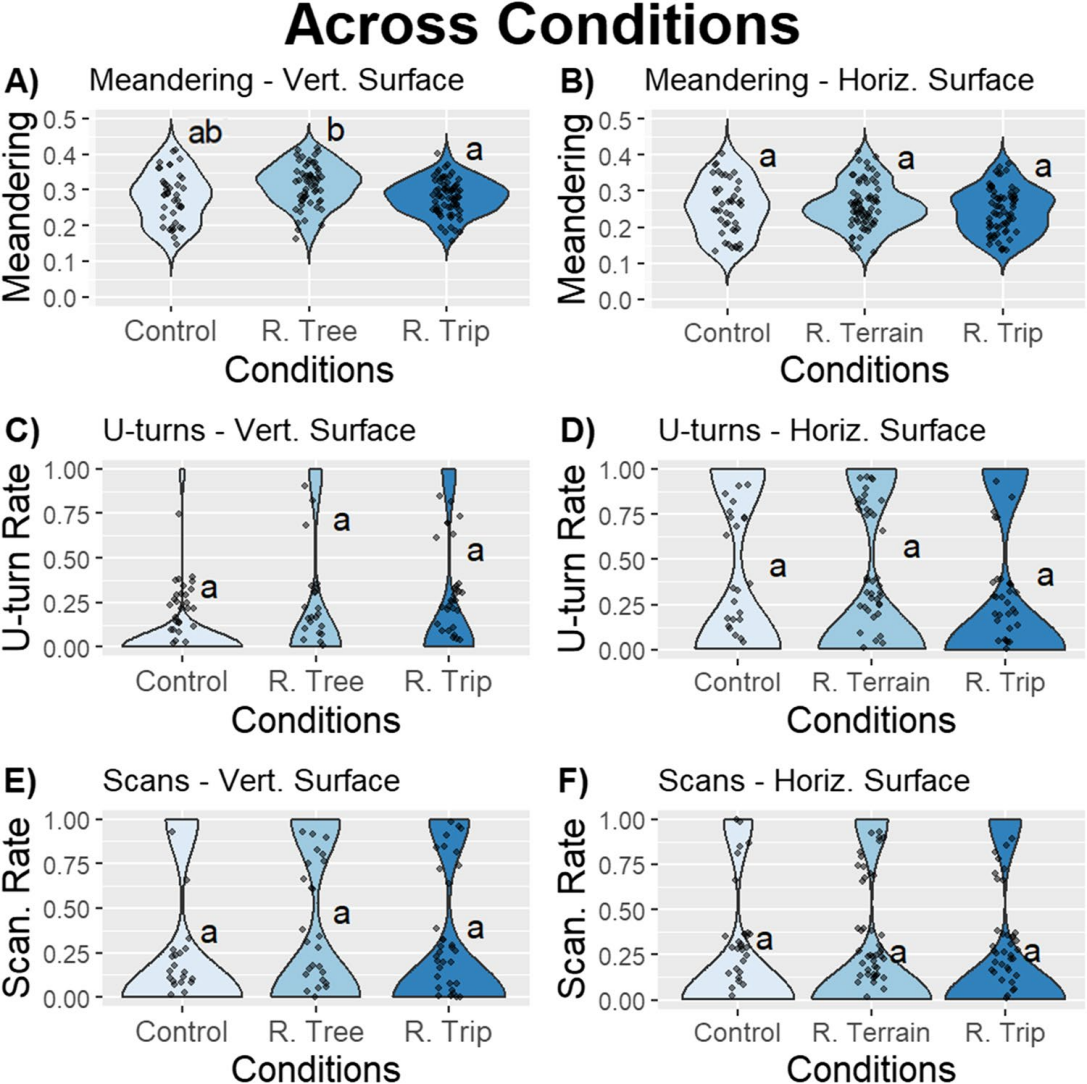
The jitter and violin plots show the meandering across conditions over all homing trips in the **A)** Vertical section and **B)** Horizontal section, the U-turn rates across conditions in the **C)** Vertical section and **D)** Horizontal section, and the scanning rates across conditions in the **E)** Vertical section and **F)** Horizontal section in Experiment 1 (*n* = 15). Conditions that do not share a letter are significantly different. The plots exhibit varying scales

We investigated the effect of rewinding along different portions on our measures of navigational behaviour, we found some effects of rewinding on the Vertical section, but no effect on the Horizontal rewinding. We found a significant effect of rewinding on meandering on the Vertical section (Model 1: Meandering; Vertical Section–Condition: effect size = 0.11, *F* = 12.69; *p* ≤ .01; Fig. 2A; Table 1). Rewinding on the foraging tree caused foragers to meander significantly more relative to those rewound across the entire corridor (post hoc comparison: Meandering–Vertical Section: contrast Rewind Tree–Rewind Trip,* t* = 3.12, *p* ≤ .01; Fig. 2A; Table S1). We also observed a trend towards increased meandering on tree rewound foragers relative to control foragers (post hoc comparison: Meandering–Vertical Section: contrast Control–Rewind Tree, *t* = –2.74, *p* = .02; Fig. 2A; Table S1). Rewinding on the Horizontal section of the foraging corridor had no significant effect (Model 2: Meandering; Horizontal Section–Condition: effect size = 0.02, *F* = 2.89; *p* = .24; Fig. 2B; Table 1). While we did not find an effect of the rewinding condition on scanning and U-turns on the Horizontal section (Model 1: U-turns; Horizontal Section–Condition: effect size = 0.29, *F* = 0.83; *p* = .66; Model 1: Scans; Vertical Section–Condition: effect size = 0.15, *F* = 3.26; *p* = .20; Model 2: Horizontal Section–Condition: effect size = 0.01, *F* = 0.27; *p* = .88; Fig. 2D–F; Tables 2 and 3), we did find a non-significant trend on the Vertical section (Model 2: U-turns; Vertical Section–Condition: effect size = 0.32, *F* = 7.10; *p* = .03; Fig. 2C; Table 2).

**Table 1:** Generalized linear mixed-models result for meandering in the Vertical and Horizontal sections in the Models 1 and 2 in Experiment 1 (alpha = 0.01)

Meandering					
Model	term	Eff. size	*DF*	*F* test	*p* value
Model 1—Vertical	Condition	0.11	2	12.69	**≤.01**
Model 1—Vertical	Capture	0.67	4	80.16	**≤.01**
Model 1—Vertical	Condition × Capture	Na	7	27.08	**≤.01**
Model 2—Horizontal	Condition	0.02	2	2.89	0.24
Model 2—Horizontal	Capture	0.82	4	72.20	**≤.01**
Model 2—Horizontal	Condition × Capture	Na	8	13.26	0.10

**Table 2:** Generalized linear mixed-models result for U-turns in the Vertical and Horizontal sections in the Models 1 and 2 in Experiment 1 (alpha = 0.01)

U-turns					
Model 1	term	Eff. size	*DF*	*F *test	*p *value
Vertical	Condition	0.32	2	7.10	.03
Vertical	Capture	0.31	4	6.82	.15
Vertical	Condition × Capture	Na	7	8.16	.32
Model 2	term	Eff. size	*DF*	*F *test	*p *value
Horizontal	Condition	0.29	2	0.83	.66
Horizontal	Capture	0.67	4	17.84	**≤.01**
Horizontal	Condition × Capture	Na	8	8.01	.43

**Table 3:** Generalized linear mixed-models result for scanning rate in the Vertical and Horizontal section in the Models 1 and 2 in Experiment 1 (alpha = 0.01)

Scanning					
Model 1	term	Eff. size	*DF*	*F *test	*p *value
Vertical	Condition	0.15	2	3.26	.20
Vertical	Capture	0.30	4	5.01	.29
Vertical	Condition × Capture	Na	7	8.99	.25
Model 2	term	Eff. size	*DF*	*F *test	*p *value
Horizontal	Condition	0.01	2	0.27	.88
Horizontal	Capture	0.46	4	2.73	.60
Horizontal	Condition × Capture	Na	8	3.11	.93

### Experiment 1: Captures

**Fig. 3:**
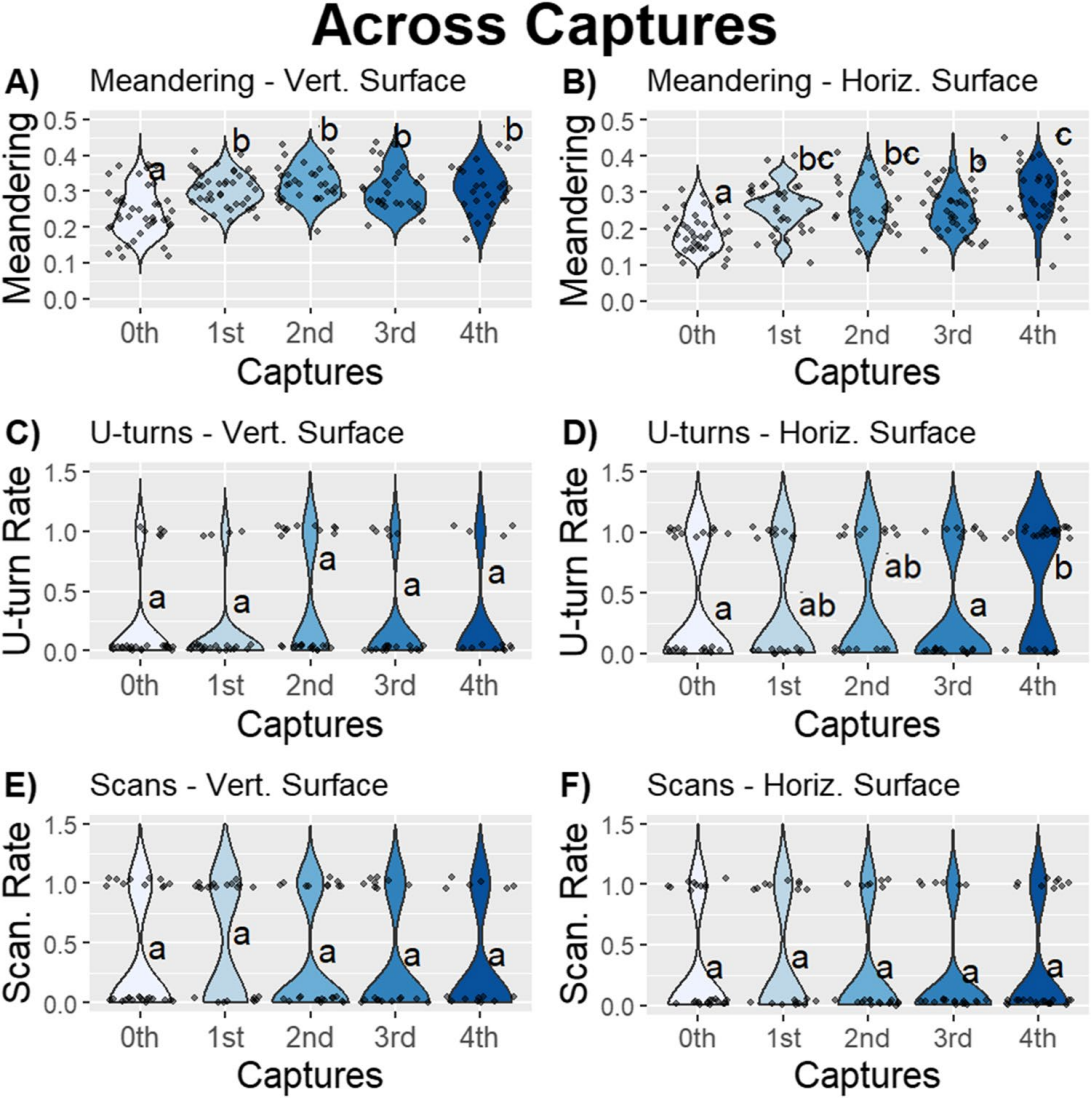
The jitter plots show the meandering over all homing trips across captures **A)** Vertical section and **B)** Horizontal section, the U-turns rates across captures in the **C)** Vertical section and **D)** Horizontal section, and the scanning rate across captures in the **E)** Vertical section and **F)** Horizontal section in Experiment 1 (*n* = 15). Captures that do not share a letter are significantly different. The plots exhibit varying scales

In contrast to the limited effect of rewinding, capturing ants had a much larger effect on forager behaviour producing more meandering and U-turns; however, it did not affect their scanning behaviour (Fig. 3; Tables 1, 2 and 3). Foragers increased meandering on both the Vertical section (Model 1: Meandering; Vertical section–Capture: effect size = 0.67, *F* = 80.16, *p* ≤ .01; post hoc comparison, contrast 0^th^–1^st^ trip; *t* = –5.66, *p* ≤ .01; Fig. 3A, Tables 1, S2), and on the Horizontal section with repeated capture-and-release procedures (Model 2: Meandering; Horizontal section–Capture: effect size = 0.82, *F* = 72.20, *p* ≤ .01; post hoc comparison, contrast 0^th^–1^st^ trip; *t* = −4.74, *p* ≤ 0.01; 3^rd^–4^th^ trip; *t* = −3.93, *p* ≤ .01; Fig. 3B; Tables 1, S2). But unlike meandering, being captured-and-released caused foragers to increase their U-turns on the Horizontal section (Model 2: U-turns; Horizontal section–Capture: effect size = 0.67,* F* = 17.84, *p* ≤ .01; post hoc comparison, Horizontal section, contrast 3^rd^–4^th^ trip; *t* = −3.68, *p* ≤ .01; Fig. 3C; Tables 2, S3), not the Vertical section (Model 1: U-turns; Vertical section–Capture: effect size = 0.31, *F* = 6.82, *p* = .15; Fig. 3D; Table 2). Capturing and releasing foragers also did not significantly impact their scanning behaviour (Model 1: Scans; Vertical section–Capture: effect size = 0.30, *F* = 5.01, *p* = .29; Model 2: Scans; Horizontal–Capture: effect size = 0.46, *F* = 2.73, *p* = .60; Fig. 3E–F; Table 3).

**Fig. 4:**
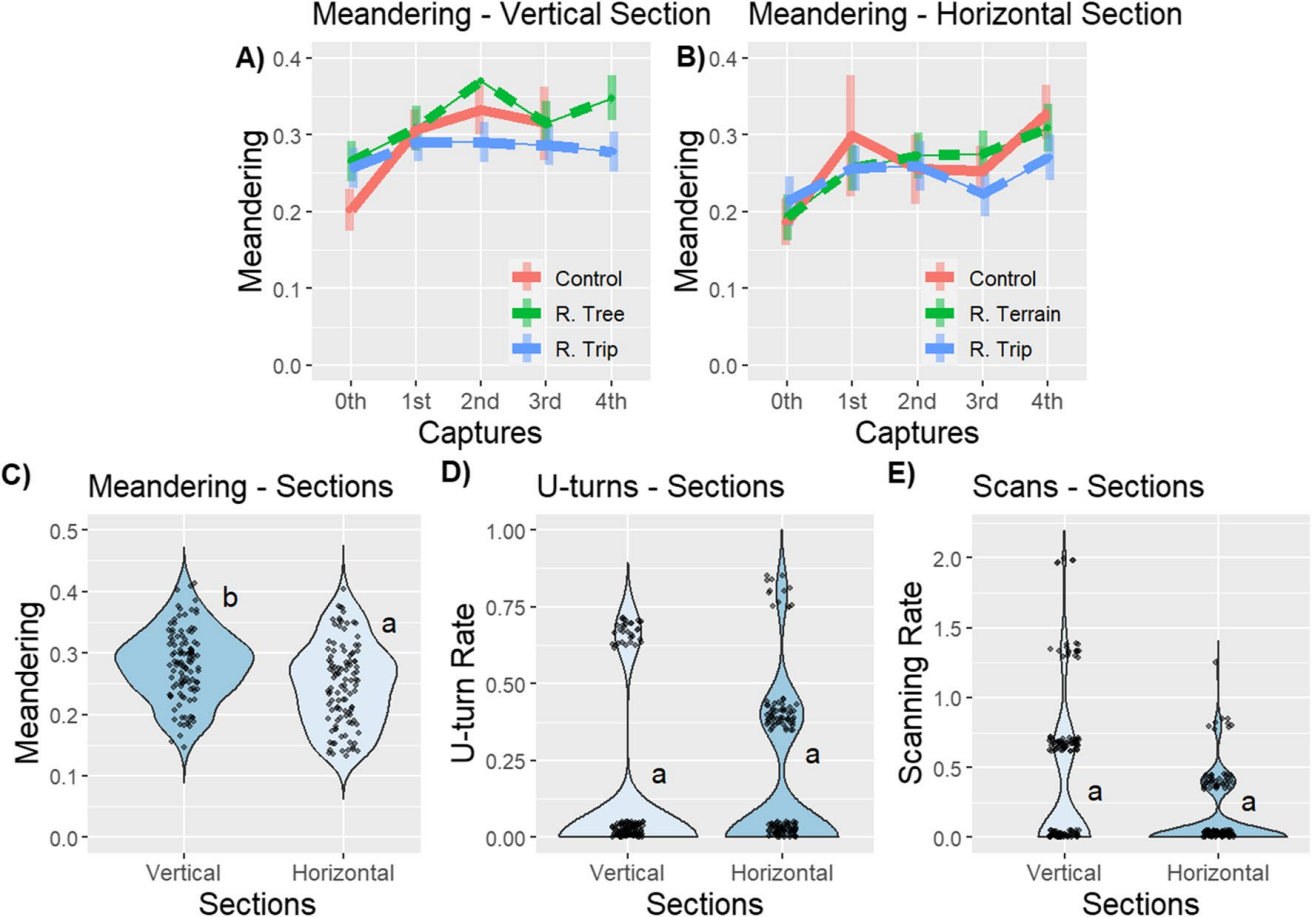
Interactions in meandering with 95% confidence intervals between Condition and Capture in the **A)** Vertical and **B)** Horizontal sections in Experiment 1. The jitter plot shows the **C)** meandering, **D)** U-turn rate, and **E)** scanning rate across sections over all homing trips in Experiment 1 (*n* = 15). Sections that do not share a letter are significantly different. The plots exhibit varying scales. (Colour figure online)

In addition to finding independent effects of Captures and Conditions on meandering, we also found an interaction between these two terms on the Vertical section (Model 1: Meandering; Vertical section: Condition × Capture, *F* = 27.08; *p* ≤ .01; Fig. 4A; Table 1), but not the Horizontal section (Model 2: Meandering; Horizontal section: Condition × Capture, *F* = 13.26; *p* = .10; Fig. 4B; Table 1). This interaction appears to be driven by increases in meandering for Tree rewound foragers relative to Trip rewound foragers after the 2^nd^ and 4^th^ capture (Meandering–Vertical Section: 2^nd^ capture, contrast Rewind Tree–Rewind Trip, *t* = 3.90, *p* ≤ .01; 4^th^ capture, contrast Rewind Tree–Rewind Trip, *t* = 3.51, *p* ≤ .01; Fig. 4A; Table S4). Together these results suggest that captures play a major role in the meandering and U-turn behaviour of these ants, but the extent of these changes are limited by vertical rewinding, and not horizontal rewinding.

### Experiment 1: Vertical and horizontal navigation

We tested whether ants behave differently when climbing vertically, than when walking horizontally regardless of rewinding and captures, and found that foragers generally exhibit more meandering while walking on a vertical surface compared to a horizontal surface, but similar U-turns and scanning behaviours (Model 3: Sections; Meandering, *F* = 9.07, *p* ≤ .01; U-turns, *F* = 2.89, *p* = .09; Scanning, *F* = 3.07, *p* = .08; post hoc comparison, contrast Horizontal section–Vertical section; *t* = −3.64, *p* ≤ .01; Fig. 4C–E; Table 4). This independent effect suggests distinct navigational challenges imposed by vertical travel.

**Table 4:** Generalized linear mixed-models result for meandering, U-turn rate, and scanning rate in the Vertical and Horizontal sections in the Model 3 in Experiment 1 (alpha = 0.01)

Vertical–Horizontal Sections				
Model	Variable	*DF*	*F* test	*p* value
Model 3	Meandering	1	9.07	**≤.01**
Model 3	U-turns	1	2.89	.09
Model 3	Scanning	1	3.07	.08

### Experiment 2: Initial response

**Fig. 5:**
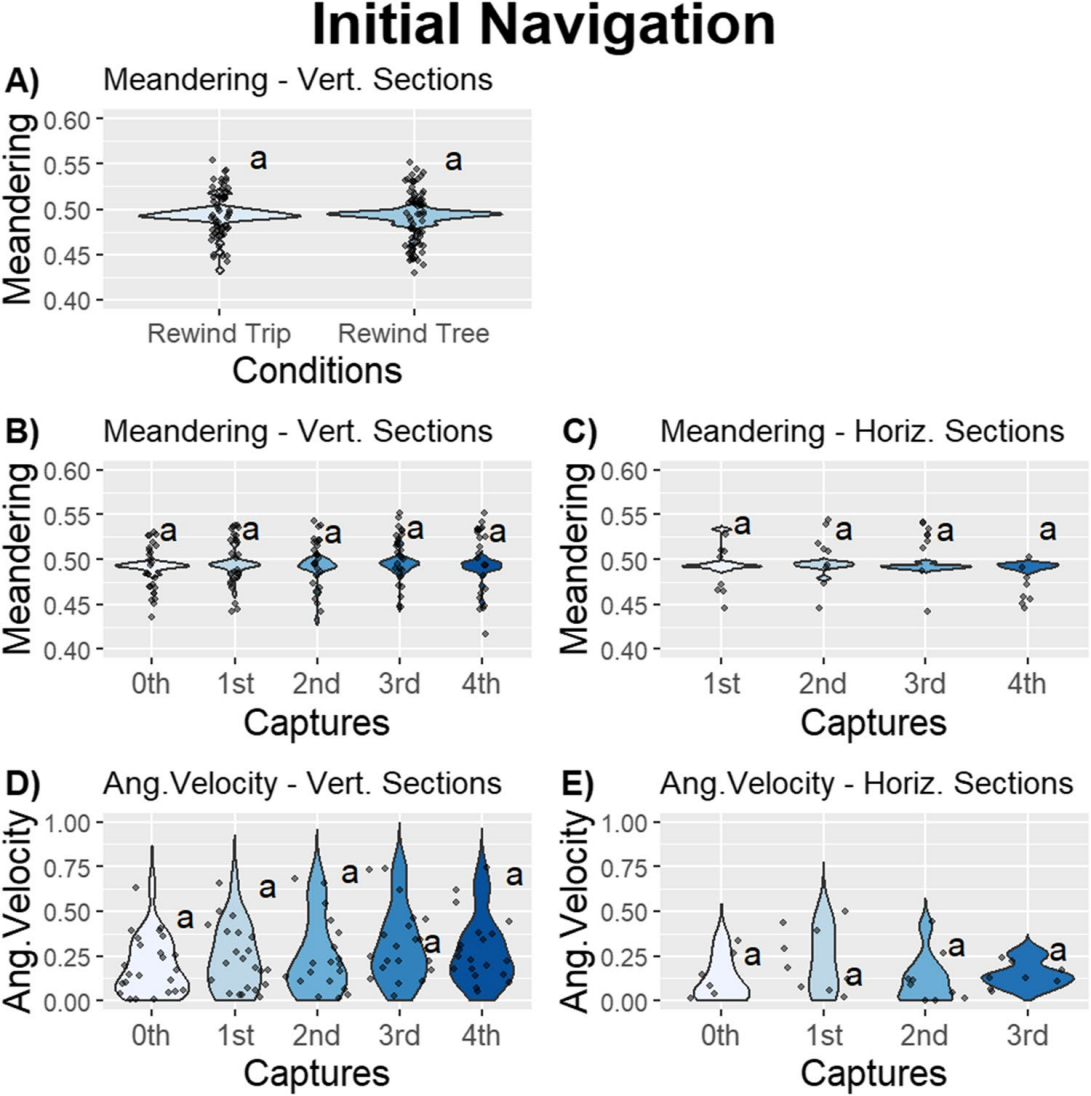
The jitter plots show **A)** the initial meandering over all homing trips between sections in the Vertical section, the initial meandering across captures in the **B)** Vertical section and **C)** Horizontal section, the initial angular velocity across captures in the **D)** Vertical section and **E)** Horizontal section in Experiment 2 (total* n* = 37). Variables that do not share a letter are significantly different. The plots exhibit varying scales

To test and investigate the foragers’ responses to the capture-and-release procedure, we investigated their initial response just after being released. We found no effect of rewinding and captures on meandering, or angular velocity in foragers’ initial behaviours when released on either a Vertical or Horizontal section. Foragers exhibited similar initial meandering and initial angular velocity when they re-experienced the whole foraging corridor or a part of it (Model 4: initial meandering; Vertical section–Condition, effect size = 0.01, *F* = 0.13; *p* = .72; initial angular velocity; Vertical section–Condition, effect size = 0.01, *F* = 0.04; *p* = .85; Fig. 5; Table 5). Foragers’ meandering and angular velocity were maintained stably over repeated captures in both Vertical and Horizontal sections (Model 4: initial meandering; Vertical section–Capture, effect size = 0.35, *F* = 2.63; *p* = .62; Model 5: initial meandering; Horizontal section–Trip, *F* = 3.14; *p* = .37; Model 4: initial angular velocity; Vertical section–Trip, effect size = 0.34, *F* = 1.23; *p* = .87; Model 5: initial angular velocity; Horizontal section–Trip, *F* = 5.05; *p* = .17; Fig. 5; Table 5). This suggests that the responses we observed in Experiment 1 are the result of some kind of escape response to being captured.

**Table 5:** Generalized linear mixed-models result for initial meandering and initial absolute angular velocity in the Vertical and Horizontal sections in the Models 4 and 5 in Experiment 2 (alpha = 0.01)

Initial Orientation						
Model	Variable	term	Eff. size	*DF*	*F* test	*p* value
Model 4–Vertical	Meandering	Condition	0.01	1	0.13	.72
Model 4–Vertical	Meandering	Capture	0.35	4	2.63	.62
Model 4–Vertical	Meandering	Condition:Capture	Na	4	4.88	.30
Model 5–Horizontal	Meandering	Capture	Na	3	3.14	.37
Model 4–Vertical	Ang. Velocity	Condition	0.01	1	0.04	.85
Model 4–Vertical	Ang. Velocity	Capture	0.34	4	1.23	.87
Model 4–Vertical	Ang. Velocity	Condition:Capture	Na	1	5.71	.22
Model 5–Horizontal	Ang. Velocity	Capture	Na	3	5.05	.17

## Discussion

When rewinding *M. midas* foragers on the Vertical and Horizontal sections of their homing trip, we found that while rewinding had a modest effect on the Vertical section, it was capturing the ants which had the largest effect on their navigational behaviour. We were unsure whether rewinding on the vertical surface would affect the navigation of these foragers since it is unknown whether they accumulate a path integration vector during vertical travel. However, surprisingly, we found that of all three categories of rewinding (vertical, horizontal, and both), only vertical rewinding caused increases in meandering, despite having the shortest rewinding vector. It was capturing and releasing the ants that had the largest effect on their meandering and U-turns, suggesting that aversive behaviours play a far larger role than path integration vectors in the navigational behaviour of experienced *M. midas* foragers. We also found an interaction between rewinding conditions and captures while descending the foraging tree, whereby Rewind Tree foragers meandered more on their 2^nd^ capture, relative to Rewind Trip foragers. Perhaps suggesting that both the location and the number of captures impact the magnitude of behavioural responses. Perhaps unsurprisingly, we also found an independent effect of surface profile, whereby foragers exhibited higher meandering when navigating vertically along the foraging tree, rather than when on the ground. It may be that walking on a vertical surface is a more challenging navigational task than walking on a horizontal surface for *M. midas* foragers, perhaps due to differences in navigational cue availability or differences in the demands of vertical locomotion.

### Effect of rewinding

*Myrmecia midas* foragers appear to ignore their path integration, despite showing some effect of vertical rewinding on the tree. We did not detect any effect of Rewind Terrain, or Rewind Trip on any measures of navigational uncertainty. Other studies rewound *M. bagoti* foragers four times for a total of 40 meters and detected an increase in meandering and U-turn behaviours (Wystrach et al., 2019), Deeti et al. (2023) rewound *M. midas* foragers and first detected increases in meandering, U-turns, and scanning behaviours after six horizontal rewinding trips, a total distance of 42 meters. While it may be that rewinding four times for a total length of 16 meters is insufficient for this species, it could also be that this species does not attend to their path integration vector when visual cues are available. Other studies have shown longer path integration vector lengths lead to stronger weighting and greater behavioural changes (Deeti et al., 2023; Freas et al., 2017a, b; Wystrach et al., 2019). And so, while we would expect Rewind Trip foragers to therefore have the greatest behavioural response, paradoxically we instead found that foragers rewound only on the Vertical section (total of 6 meters) meandered more than those rewound on both Vertical and Horizontal sections. Given that Rewind Trip foragers were also rewound on the vertical section, this increase in meandering does not appear to be due to these ants only accumulating vertical vectors. Since these ants do not appear to be attending to vectors, whether vertical or horizontal, this increase in meandering may be due to an increased sensitivity to interference while on the tree relative to ground travel, perhaps because they are at a higher risk of predation on the higher contrast tree surface. Overall, we conclude that unlike some species, *M. midas* foragers make minimal use of their path integration vector while foraging, and instead rely on visual cues to find their way home.

### Effect of captures

The effect of being captured and released had a larger effect on forager meandering and U-turns than being rewound. *Myrmecia midas* foragers increased their meandering on both horizontal and vertical portions of the foraging corridor, with each successive capture. Foragers also increased their U-turning rate in the horizontal portion after being captured and released. This result is consistent with recent work in this species where foragers showed aversive responses, including location avoidance, after being subject to a series of capture-and-releases at a specific location during their foraging and homing trips (Lionetti et al., 2023). However, this is in contrast to desert ants like *M. bagoti* and *C. fortis* which are unaffected by similar, and even more extreme physical manipulations (Freas & Cheng, 2018; Wittlinger et al., 2006; Wystrach et al., 2019). Given that *M. midas* shows a greater sensitivity to physical manipulations than desert ants, it appears that the effect of capture overshadows any effect that a mismatched path integration vector may have (Lionetti et al., 2023). Regardless, it remains surprising that the magnitude of the effect of capture was so much higher than the effect of rewinding.

### Measures of navigational behaviour

Increases in meandering, U-turns, and scanning behaviours are all common responses to rewinding (Deeti et al., 2023; Wystrach et al., 2019), and while we found an effect of being rewound on meandering when walking on a vertical surface, its effect on U-turns was a non-significant trend, and there was no change in scanning across any treatment. The meandering of these foragers reached an asymptote by the second trip, as further captures and rewinding did not lead to higher meandering. Such an asymptote contrasts with *M. bagoti* foragers which showed increasing meandering, and U-turns over four repeated rewinding procedures (Wystrach et al., 2019) and in contrast to increasing meandering, U-turns and scans in the previous *M. midas*’s horizontal rewinding experiment (Deeti et al., 2023). We also found weak evidence that U-turns increased due to vertical rewinding and full trip rewinding relative to captures on this horizontal portion of the trip. While this may suggest that foragers are accumulating vertical path integration vectors, captures did not prompt U-turns in the vertical portion of the trip, so it may just reflect that U-turns are rare while on the tree. Given the propensity of other species to perform U-turns, we were surprised that we did not see a similar response in *M. midas* (Deeti et al., 2023; Wystrach et al., 2019). The lack of change in scanning rates is also surprising since increases in scanning were even observed in previous rewinding experiments on *M. midas* (Deeti et al., 2023). However, in Deeti et al.’s (2023) experiment, the foragers had a homing path of 7 meters and were rewound nine times, compared to the 2.5 meters vector of our Rewind Terrain foragers and 4 meters for our Rewind Trip foragers. Shorter rewinding distances mean shorter path integration vectors and previous work has suggested that longer vectors provoke stronger responses in terms of weightings or preferences during navigation (Hoinville & Wehner, 2018; Wystrach et al., 2015). The differences we observe in this work could be due to the difference in vector length, which could lead to foragers having a weaker conflict between path integration and terrestrial cues, triggering a lower number of scans. As such, this experiment may not have crossed the path integration length threshold necessary to observe changes in behaviour in this species. The minimal impact of rewinding we observe in this experiment may be due to *M. midas* foragers’ limited use of path integration (Freas et al., 2017a, b), their shorter path integration vector (Hoinville & Wehner, 2018; Wystrach et al., 2015), or their sensitivity to physical manipulations (Lionetti et al., 2023). The limited use of path integration might be due to differences in the foraging strategies of *M. midas* compared with desert ants, since *M. bagoti* and *C. fortis* foraging strategies involve scouting for a food source, whereas *M. midas*’s foraging trees are stationary (Freas et al., 2018).

We found that the first visual repetition does not trigger an increase in scanning, which is suggestive of learning behaviour in *M. midas* foragers. Ants are known to scan more frequently when they are engaging in learning tasks (Deeti & Cheng, 2021; Fleischmann et al., 2017). We found no evidence that the rewinding procedure caused the foragers to increase their number of scans. These results are in contrast with *M. bagoti* foragers, which showed an increasing number of scans over repeated rewinding procedures (Wystrach et al., 2019). Foragers performed more scans when facing unfamiliar or unexpected views (Baddeley et al., 2011; Philippides et al., 2011; Wystrach et al., 2014). Here, *M. midas* foragers seem to disregard or be unaware of reexperiencing previous views. It remains unclear why repeated visual repetitions do not trigger foragers’ scanning behaviour. We speculate that the view unfamiliarity itself triggers an increase in foragers’ scanning behaviour, with foragers seeming to perceive the paradox of visual repetition due to the rewinding procedure.

### Vertical versus horizontal locomotion

Foragers exhibit higher meandering when walking on the vertical segment of the foraging corridor compared to the horizontal segment, but not more U-turns or scanning. This could suggest that vertical locomotion is more challenging, that vertical navigation is harder, or that foragers are more cautious while on the tree-trunk surface. While many ant species spend the majority of their lives climbing trees (Nadkarni, 1994), there remain differences in locomotion as foragers cannot rely on gravity to keep them bound to the tree’s surface, as they can while on the ground. Differences in the views available while on the tree surface may also impose challenges, as studies have suggested walking animals perceive vertical spatial information less accurately (Nuri Flores-Abreu et al., 2014). While *Myrmecia* ants stabilize their head to varying degrees against locomotion-induced body roll movements (Raderschall et al., 2016), it is not always practical to do so, especially when climbing or descending a tree. *Myrmecia midas* foragers have been shown to use surrounding views to correctly orient towards the tree side facing the nest while climbing/descending the tree (Freas et al., 2018). As such, any difficulties in navigation could be due to differences in the structure of vertically and horizontally aligned views, or due to the involvement of other sensory systems, perhaps gravitational or geomagnetic. It is also possible that like the many species which have been shown to monitor their risk of detection by a predator and adjust their behaviour (Apfelbach et al., 2005), these ants could be attempting to evade predation. The tree trunk is often also the highest contrast surface that these ants cross during their navigation, as such, these ants could be meandering more, so as to increase their ability to detect and avoid threats. It is unclear at this point whether this difference in meandering was due to locomotion, navigation, or threat avoidance, however, it appears that understanding the unique challenges of vertical navigation is a fruitful area for future ant navigational research.

### Initial navigation

When we investigated the initial portions of the trip, we did not find any effect of rewinding or captures on any of our measures of behaviour. This lack of difference could be due to their immediate aversive response to being captured or could be due to an inability to detect differences in these variables over these smaller time scales. We have established that capture causes characteristic changes in forager behaviour, and it could be that immediately on release, this response overshadows any other factors, leading to a similar aversive reaction after each capture-and-release procedure. It is also possible that the size and duration of the recording window are too small to capture enough of the extent of these behavioural changes to be detectable. Future experiments that investigate initial portions of navigational behaviour could record over larger areas; however, there are trade-offs between resolution and area to consider. It may be more fruitful instead to ensure good positive and negative controls for the effects of capture in any experiments involving longer-lived species, while also pursuing explicit tests for such effects, in coordination with comparative work on aversive responses.

## Conclusion

We found that *M. midas* foragers can successfully find their way home even after undergoing multiple rewinding procedures; however, the manipulation had a disruptive effect on navigational behaviours. Rewound foragers do not seem to accumulate path integration vector. In comparison, being captured-and-released led to large increases foragers’ meandering and U-turns, but not their scanning behaviours. The current study also found that foragers exhibited higher meandering when walking on the foraging tree, suggesting that vertical navigation may be more challenging than horizontal navigation for *M. midas* foragers. Overall, we see large differences in *M. midas*’s response to the rewinding procedure relative to desert ants such as *M. bagoti*, these changes appear due to *M. midas* foragers’ minimal reliance on path integration in navigation and higher sensitivity to physical manipulation.

### Supplementary Information

Supplementary videos and data are available at Open Science Framework: https://osf.io/ag5eb. Below is the link to the electronic supplementary material.

Supplementary file1 (DOCX 376 KB)

## Data Availability

https://osf.io/ag5eb.

## References

[CR1] Andel D, Wehner R (2004). Path integration in desert ants, *Cataglyphis*: How to make a homing ant run away from home. Proceedings of the Royal Society of London Series B: Biological Sciences.

[CR2] Apfelbach R, Blanchard CD, Blanchard RJ, Hayes RA, McGregor IS (2005). The effects of predator odors in mammalian prey species: A review of field and laboratory studies. Neuroscience & Biobehavioral Reviews.

[CR3] Baddeley B, Graham P, Philippides A, Husbands P (2011). Holistic visual encoding of ant-like routes: Navigation without waypoints. International Society for Adaptive Behavior.

[CR4] Benhamou S (2004). How to reliably estimate the tortuosity of an animal's path: straightness, sinuosity, or fractal dimension?. Journal of Theoretical Biology.

[CR5] Bühlmann C, Cheng K, Wehner R (2011). Vector-based and landmark-guided navigation in desert ants inhabiting landmark-free and landmark-rich environments. Journal of Experimental Biology.

[CR6] Cheng K, Narendra A, Sommer S, Wehner R (2009). Traveling in clutter: Navigation in the Central Australian desert ant *Melophorus bagoti*. Behavioural Processes.

[CR7] Cheng K, Schultheiss P, Schwarz S, Wystrach A, Wehner R (2014). Beginnings of a synthetic approach to desert ant navigation. Behavioural Processes.

[CR8] Collett M (2012). How navigational guidance systems are combined in a desert ant. Current Biology.

[CR9] Collett, M. (2014). A desert ant’s memory of recent visual experience and the control of route guidance. *Proceedings of the Royal Society B: Biological Sciences, 281*(1787). 10.1098/RSPB.2014.0634 10.1098/rspb.2014.0634 PMC4071549 24870046

[CR10] Deeti, S., & Cheng, K. (2021). Learning walks in an Australian desert ant, *Melophorus bagoti.*10.1242/jeb.242177 10.1242/jeb.242177 PMC8407660 34435625

[CR11] Deeti S, Islam M, Freas C, Murray T, Cheng K (2023). Intricacies of running a route without success in night-active bull ants (*Myrmecia midas*). Journal of Experimental Psychology: Animal Learning and Cognition.

[CR12] Fleischmann PN, Grob R, Wehner R, Rössler W (2017). Species-specific differences in the fine structure of learning walk elements in *Cataglyphis* ants. Journal of Experimental Biology.

[CR13] Freas CA, Cheng K (2017). Learning and time-dependent cue choice in the desert ant *Melophorus bagoti*. Ethology.

[CR14] Freas CA, Cheng K (2018). Landmark learning, cue conflict, and outbound view sequence in navigating desert ants. Journal of Experimental Psychology: Animal Learning and Cognition.

[CR15] Freas CA, Narendra A, Cheng K (2017). Compass cues used by a nocturnal bull ant, *Myrmecia midas*. Journal of Experimental Biology.

[CR16] Freas, C. A., Narendra, A., Lemesle, C., & Cheng, K. (2017). Polarized light use in the nocturnal bull ant, *Myrmecia midas*. *Royal Society Open Science, 4*(8). 10.1098/RSOS.170598 10.1098/rsos.170598 PMC5579118 28879002

[CR17] Freas CA, Wystrach A, Narendra A, Cheng K (2018). The view from the trees: Nocturnal bull ants, *Myrmecia midas*, use the surrounding panorama while descending from trees. Frontiers in Psychology.

[CR18] Heinze S, Narendra A, Cheung A (2018). Principles of insect path integration. Current Biology: CB.

[CR19] Hoinville T, Wehner R (2018). Optimal multiguidance integration in insect navigation. Proceedings of the National Academy of Sciences of the United States of America.

[CR20] Islam M, Deeti S, Mahmudah Z, Kamhi JF, Cheng K (2023). Detouring while foraging up a tree: What bull ants (*Myrmecia midas*) learn and their reactions to novel sensory cues. Journal of Comparative Psychology.

[CR21] Legge ELG, Wystrach A, Spetch ML, Cheng K (2014). Combining sky and earth: Desert ants (*Melophorus bagoti*) show weighted integration of celestial and terrestrial cues. Journal of Experimental Biology.

[CR22] Lionetti VAG, Deeti S, Murray T, Cheng K (2023). Resolving conflict between aversive and appetitive learning of views: How ants shift to a new route during navigation. Learning & Behavior.

[CR23] Mathis A, Mamidanna P, Cury KM, Abe T, Murthy VN, Mathis MW, Bethge M (2018). DeepLabCut: Markerless pose estimation of user-defined body parts with deep learning. Nature Neuroscience.

[CR24] Nadkarni NM (1994). Diversity of species and interactions in the upper tree canopy of forest ecosystems. Integrative and Comparative Biology.

[CR25] Narendra A (2007). Homing strategies of the Australian desert ant Melophorus bagoti. Interaction of the path integrator with visual cue information. Journal of Experimental Biology.

[CR26] Narendra, A., Gourmaud, S., & Zeil, J. (2013). Mapping the navigational knowledge of individually foraging ants, *Myrmecia croslandi*. *Proceedings of the Royal Society B: Biological Sciences, 28*0(1765). 10.1098/RSPB.2013.0683 10.1098/rspb.2013.0683 PMC3712440 23804615

[CR27] Nath T, Mathis A, Chen AC, Patel A, Bethge M, Mathis MW (2019). Using DeepLabCut for 3D markerless pose estimation across species and behaviors. Nature Protocols.

[CR28] Nuri Flores-Abreu, I., Hurly, T. A., Ainge, J. A., & Healy, S. D. (2014). Three-dimensional space: Locomotory style explains memory differences in rats and hummingbirds. *Proceedings of the Royal Society B: Biological Sciences, 281*(1784). 10.1098/RSPB.2014.0301 10.1098/rspb.2014.0301 PMC4043095 24741019

[CR29] Philippides A, Baddeley B, Cheng K, Graham P (2011). How might ants use panoramic views for route navigation?. Journal of Experimental Biology.

[CR30] R Core Team (2021). R: A language and environment for statistical computing. R Foundation for Statistical Computing, Vienna, Austria. https://www.R-project.org/

[CR31] Raderschall CA, Narendra A, Zeil J (2016). Head roll stabilisation in the nocturnal bull ant *Myrmecia pyriformis*: Implications for visual navigation. Journal of Experimental Biology.

[CR32] Ronacher B (2020). Path integration in a three-dimensional world: The case of desert ants. Journal of Comparative Physiology A: Neuroethology, Sensory, Neural, and Behavioral Physiology.

[CR33] Shapiro SS, Wilk AMB (1965). An analysis of variance test for normality. Biometrika.

[CR34] Stone T, Webb B, Adden A, Scimeca L, Warrant E, Correspondence SH (2017). An anatomically constrained model for path integration in the bee brain. Current Biology.

[CR35] Warrant E, Dacke M (2010). Vision and visual navigation in nocturnal insects. Annual Reviews.

[CR36] Webb, B. (2019). The internal maps of insects. *Journal of Experimental Biology, 222*(Suppl. 1). 10.1242/JEB.188094/2809 10.1242/jeb.188094 30728234

[CR37] Wehner R, Hoinville T, Cruse H, Cheng K (2016). Steering intermediate courses: Desert ants combine information from various navigational routines. Journal of Comparative Physiology A: Neuroethology, Sensory, Neural, and Behavioral Physiology.

[CR38] Wehner R, Wehner S (1986). Path integration in desert ants. Approaching a long-standing puzzle in insect navigation. Monitore Zoologico Italiano-Italian Journal of Zoology.

[CR39] Wittlinger M, Wehner R, Wolf H (2006). The ant odometer: Stepping on stilts and stumps. Science.

[CR40] Wystrach A, Buehlmann C, Schwarz S, Cheng K, Graham P (2020). Rapid aversive and memory trace learning during route navigation in desert ants. Current Biology.

[CR41] Wystrach, A., Mangan, M., & Webb, B. (2015). Optimal cue integration in ants*. Proceedings of the Royal Society B: Biological Sciences*, *282*(1816). 10.1098/RSPB.2015.1484 10.1098/rspb.2015.1484 PMC4614770 26400741

[CR42] Wystrach A, Philippides A, Aurejac A, Cheng K, Graham P (2014). Visual scanning behaviours and their role in the navigation of the Australian desert ant *Melophorus bagoti*. Journal of Comparative Physiology A: Neuroethology, Sensory, Neural, and Behavioral Physiology.

[CR43] Wystrach A, Schwarz S, Graham P, Cheng K (2019). Running paths to nowhere: Repetition of routes shows how navigating ants modulate online the weights accorded to cues. Animal Cognition.

