# Supplementary material for: Effect of repetition of vertical and horizontal routes on navigation performance in Australian bull ants
Source: Learn Behav. 2023 Dec 5;52(1):92–104. doi: 10.3758/s13420-023-00614-z (PMC10923747; doi:10.3758/s13420-023-00614-z)
Supplement: Supplementary file 1 — Supplementary file1 (DOCX 376 KB) [file 13420_2023_614_MOESM1_ESM.docx]

## Supplementary


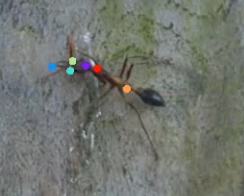


Figure S1: Figure of the extracted forager’s 6 body positions: mandible tips, left compound eye, right compound eye, back head, front thorax, and abdominal petiole.


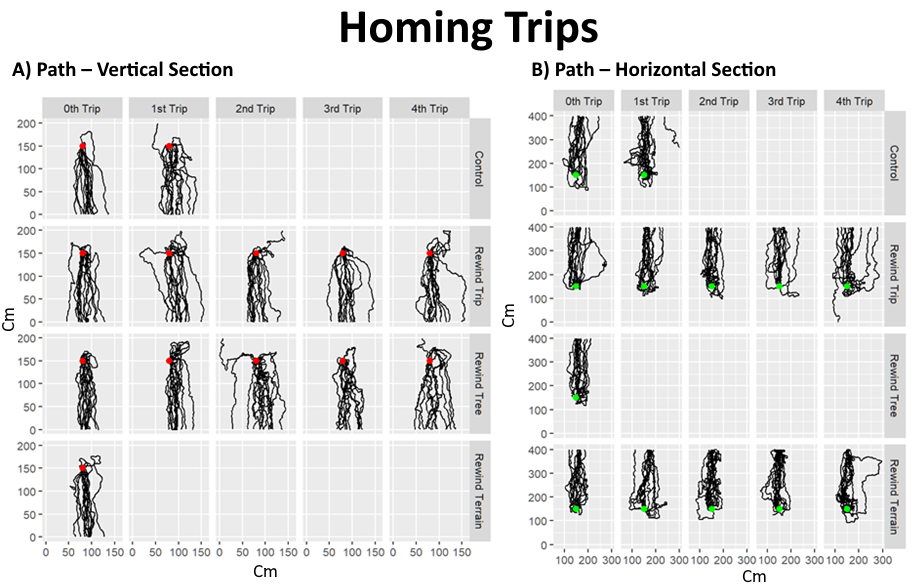


Figure S2: Individual paths of the foragers in the Control, Rewind Trip, Rewind Tree, and Rewind Terrain Conditions in the A) Vertical and B) Horizontal section (n = 15). The black lines represent the foragers’ paths. The plots exhibit varying scales. The red dot represents the Starting point. The green dot represents the nest location.

Table S1: Tukey post hoc comparisons of statistical results for meandering in the Vertical and Horizontal sections across conditions in Experiment 1 (alpha = 0.01).

| **Meandering** | | | | | | |
| --- | --- | --- | --- | --- | --- | --- |
| **Section** | **contrast** | **estimate** | **SE** | **DF** | **t ratio** | **p value** |
| Vertical | Control–Rewind Tree | –0.03 | 0.01 | 50.61 | –2.74 | 0.02 |
| Vertical | Control–Rewind Trip | 0.00 | 0.01 | 46.98 | –0.02 | 0.99 |
| Vertical | Rewind Tree–Rewind Trip | 0.03 | 0.01 | 32.38 | 3.12 | **≤ 0.01** |
| Horizontal | Control–Rewind Terrain | –0.01 | 0.01 | 49.16 | –0.47 | 0.88 |
| Horizontal | Control–Rewind Trip | 0.01 | 0.01 | 50.15 | 0.70 | 0.76 |
| Horizontal | Rewind Terrain–Rewind Trip | 0.02 | 0.01 | 32.78 | 1.34 | 0.38 |

Table S2: Tukey post hoc comparisons of statistical results for meandering in the Vertical and Horizontal sections across captures in Experiment 1 (alpha = 0.01).

| **Meandering** | | | | | | |
| --- | --- | --- | --- | --- | --- | --- |
| **Section** | **Capture** | **estimate** | **SE** | **DF** | **t ratio** | **p value** |
| Vertical | 0–1 | –0.06 | 0.01 | 125.63 | –5.66 | **≤ 0.01** |
| Vertical | 0–2 | –0.08 | 0.01 | 128.72 | –7.59 | **≤ 0.01** |
| Vertical | 0–3 | –0.06 | 0.01 | 128.78 | –5.08 | **≤ 0.01** |
| Vertical | 0–4 | –0.07 | 0.01 | 130.73 | –5.37 | **≤ 0.01** |
| Vertical | 1–2 | –0.03 | 0.01 | 128.99 | –2.23 | 0.17 |
| Vertical | 1–3 | 0.00 | 0.01 | 132.13 | 0.05 | 0.99 |
| Vertical | 1–4 | –0.01 | 0.01 | 132.67 | –0.48 | 0.99 |
| Vertical | 2–3 | 0.03 | 0.01 | 129.03 | 2.11 | 0.22 |
| Vertical | 2–4 | 0.02 | 0.01 | 128.64 | 1.51 | 0.56 |
| Vertical | 3–4 | –0.01 | 0.01 | 126.43 | –0.50 | 0.99 |
| Horizontal | 0–1 | –0.06 | 0.01 | 144.03 | –4.74 | **≤ 0.01** |
| Horizontal | 0–2 | –0.07 | 0.01 | 143.31 | –5.15 | **≤ 0.01** |
| Horizontal | 0–3 | –0.05 | 0.01 | 140.49 | –4.37 | **≤ 0.01** |
| Horizontal | 0–4 | –0.10 | 0.01 | 140.96 | –8.16 | **≤ 0.01** |
| Horizontal | 1–2 | 0.00 | 0.01 | 139.94 | –0.35 | 0.99 |
| Horizontal | 1–3 | 0.01 | 0.01 | 142.42 | 0.67 | 0.96 |
| Horizontal | 1–4 | –0.04 | 0.01 | 143.22 | –2.99 | 0.03 |
| Horizontal | 2–3 | 0.01 | 0.01 | 141.44 | 1.05 | 0.83 |
| Horizontal | 2–4 | –0.04 | 0.01 | 142.18 | –2.65 | 0.07 |
| Horizontal | 3–4 | –0.05 | 0.01 | 135.89 | –3.93 | **≤ 0.01** |

Table S3: Tukey post hoc comparisons of statistical results for U-turns in the Horizontal sections across captures in Experiment 1 (alpha = 0.01).

| **U-turns - Horizontal section** | | | | | |
| --- | --- | --- | --- | --- | --- |
| **Capture** | **estimate** | **SE** | **DF** | **t ratio** | **p value** |
| 0–1 | 0.00 | 0.05 | 148.98 | –0.05 | 0.99 |
| 0–2 | –0.05 | 0.05 | 150.99 | –0.91 | 0.89 |
| 0–3 | 0.02 | 0.05 | 143.70 | 0.33 | 0.99 |
| 0–4 | –0.17 | 0.05 | 144.93 | –3.43 | **≤ 0.01** |
| 1–2 | –0.05 | 0.06 | 147.95 | –0.81 | 0.93 |
| 1–3 | 0.02 | 0.05 | 149.41 | 0.35 | 0.99 |
| 1–4 | –0.17 | 0.05 | 149.36 | –3.10 | 0.02 |
| 2–3 | 0.06 | 0.05 | 150.18 | 1.19 | 0.76 |
| 2–4 | –0.12 | 0.06 | 151.69 | –2.18 | 0.19 |
| 3–4 | –0.19 | 0.05 | 143.32 | –3.68 | **≤ 0.01** |

Table S4: Tukey post hoc comparisons of statistical results for meandering in the Vertical section across captures in the different conditions in Experiment 1 (alpha = 0.01).

| **Meandering** | | | | | | |
| --- | --- | --- | --- | --- | --- | --- |
| **Capture** | **contrast** | **estimate** | **SE** | **DF** | **t ratio** | **p value** |
| 1 | Control - Rewind Tree | 0.00 | 0.02 | 139.52 | –0.12 | 0.99 |
| 1 | Control - Rewind Trip | 0.02 | 0.02 | 132.38 | 0.87 | 0.66 |
| 1 | Rewind Tree - Rewind Trip | 0.02 | 0.02 | 138.47 | 0.94 | 0.62 |
| 2 | Control - Rewind Tree | –0.04 | 0.02 | 147.30 | –1.66 | 0.22 |
| 2 | Control - Rewind Trip | 0.04 | 0.02 | 142.83 | 2.00 | 0.12 |
| 2 | Rewind Tree - Rewind Trip | 0.08 | 0.02 | 142.15 | 3.90 | **≤ 0.01** |
| 3 | Control - Rewind Tree | 0.00 | 0.03 | 153.47 | 0.00 | 0.99 |
| 3 | Control - Rewind Trip | 0.03 | 0.03 | 153.16 | 1.01 | 0.57 |
| 3 | Rewind Tree - Rewind Trip | 0.03 | 0.02 | 139.60 | 1.40 | 0.34 |
| 4 | Control - Rewind Tree | Na | Na | Na | Na | Na |
| 4 | Control - Rewind Trip | Na | Na | Na | Na | Na |
| 4 | Rewind Tree - Rewind Trip | 0.07 | 0.02 | 139.49 | 3.51 | **≤ 0.01** |
